# Structured content and data management—enhancing acceleration in drug development through efficiency in data exchange

**DOI:** 10.1186/s41120-023-00077-6

**Published:** 2023-05-08

**Authors:** Jill Beierle, Marquerita Algorri, Marisol Cortés, Nina S. Cauchon, Andrew Lennard, J. Paul Kirwan, Shirley Oghamian, Michael J. Abernathy

**Affiliations:** 1 Department of Global Regulatory Affairs and Strategy – CMC, Amgen Inc, CA 91320 Thousand Oaks, USA; 2 Department of Physiology, Johns Hopkins University School of Medicine, Baltimore, MD 21205 USA

**Keywords:** Regulatory science, Standards, Structure, Stability, Automation, Acceleration pathway, Chemistry manufacturing and controls, Structured content data management, Cloud-based systems

## Abstract

Innovation in pharmaceutical therapeutics is critical for the treatment of serious diseases with unmet medical need. To accelerate the approval of these innovative treatments, regulatory agencies throughout the world are increasingly adopting the use of expedited pathways and collaborative regulatory reviews. These pathways are primarily driven by promising clinical results but become challenging for Chemistry, Manufacturing, and Controls (CMC) information in regulatory submissions. Condensed and shifting timelines present constraints that require new approaches to the management of regulatory filings. This article emphasizes technological advances that have the potential to tackle the underlying inefficiencies in the regulatory filing eco-system.

Structured content and data management (SCDM) is highlighted as a foundation for technologies that can ease the burden on both sponsors and regulators by streamlining data usage in regulatory submissions. Re-mapping of information technology infrastructure will improve the usability of data by moving away from document-based filings towards electronic data libraries. Although the inefficiencies of the current regulatory filing eco-system are more evident for products that are filed using expedited pathways, it is envisioned that the more widespread adoption of SCDM, across standard filing and review processes, will improve overall efficiency and speed in the compilation and review of regulatory submissions.

## Introduction

The pharmaceutical industry is complex and highly regulated, since global health authorities and biopharmaceutical companies are mutually responsible for ensuring that marketed pharmaceutical products are safe and efficacious. While thorough regulatory review and approval processes are necessary in order to ensure product safety, efficacy, and quality, the staggered and prolonged timelines required for global approvals contribute to delays in patient access to lifesaving medicines [1–3]. To expedite product availability for indications with unmet medical need or serious illness, accelerated regulatory approval pathways have been established in several different regions.

While these expedited regulatory pathways can result in accelerated filings and, in some instances, faster review/approval timelines, they require significant resources, coordination, and program management for both regulators and sponsors. The use of these pathways is often based on promising results from clinical development but poses significant challenges for Chemistry, Manufacturing, and Controls (CMC) development and data in regulatory submissions [4–6]. For many regions, the current regulatory process requires that CMC data be supplied primarily in Module 3 of the common technical document (CTD) or electronic (e)CTD, which consists of an XML backbone structure that primarily houses PDF documents containing data, descriptions, reports, and relevant certifications. The process of compiling and maintaining CMC information and data in document format requires substantial manual input and rework throughout a product’s lifecycle, which is particularly problematic for products undergoing accelerated development with multiple simultaneous submissions at varying stages of development. Enhancing data management efficiency for regulatory submissions can help alleviate delays and reduce data entry errors by streamlining repetitive authoring tasks, structuring information to allow for automation, and enabling data-driven submission assembly and review [7].

Towards this goal, the last few decades have fostered significant technological advancements within the pharmaceutical industry, such as the International Society of Pharmaceutical Engineering (ISPE) Pharma 4.0™ which includes incorporation of technologies including cloud computing, big data analytics, Internet of Things (IoT), and artificial intelligence (AI), while supporting regulatory best practices [8–10]. While Pharma 4.0 encompasses a transformative model of manufacturing enabled by modern technology which complements Quality by Design (QbD) principles [11], in general, the pharmaceutical industry has been slow to adopt the necessary technological advancements in information systems that enable modernization of information exchange and data management. Structured content and data management (SCDM), which allows information to be modularized and reused across a centrally managed content repository, has emerged as a tool which provides a potential solution to target many of the efficiency challenges for CMC documents that persist during regulatory submission and review processes [7, 12]. While there is presently an abundance of cautionary thinking within the pharmaceutical and biopharmaceutical industry with regard to investment in novel information management technologies, the value of SCDM and Pharma 4.0 enabling approaches will continue to increase as digital industrialization continues and demonstrates the utility of rapid information sharing as a tool for increasing efficiency in regulatory submissions [13, 14]. Additionally, resistance to leverage twenty-first century technologies hinges on our industry being highly regulated. However, the banking industry is also highly regulated, yet most global monetary transactions utilize similar technologies.

This article intends to highlight how SCDM and associated technologies can work synergistically with fast-tracked products to help address the regulatory CMC challenges inherent in an accelerated development environment. A summary of expedited regulatory pathways and collaborative review processes is provided with discussion on their specific challenges. Information on key advancements in technologies that are supportive of SCDM in regulatory submissions is also summarized. A hypothetical case study, representative of a fictional filing scenario, is presented to demonstrate how SCDM could potentially be applied to meet the challenges associated with expedited product filings at various stages in development. Lastly, the future outlook is discussed.

## Expedited regulatory pathways improve patient access to new medicines

The decision to pursue an expedited review pathway with a particular health authority is typically associated with success in early-stage clinical development trials and demands earlier regulatory agency engagement in the development program as well as alignment on CMC considerations and filing expectations [15, 16]. Notably, novel modalities or therapies will need added collaborative discussions across quality, clinical, and non-clinical disciplines within health agencies. A barrier to speedy access to new medicines for patients globally is the lack of harmonization in regulatory requirements, including those that impact accelerated filing pathways. Achieving an expedited approval designation in one country does not guarantee that it will be granted elsewhere [17]. A summary of accelerated review pathways in several major markets is presented below.

The need to accelerate the availability of new therapeutics was enacted in the United States (US) in 1988 by the Food and Drug Administration (FDA) with interim regulatory procedures intended to expedite the development and review of new drugs to address unmet medical needs in treating serious or life-threatening conditions (21 CFR 312) [18]. Since then, the FDA has adopted five programs: Fast Track Designation, Breakthrough Therapy Designation (BTD), Accelerated Approval, Priority Review, and Regenerative Medicine Advanced Therapy (RMAT) [19]. Both BTD and RMAT are intended for drugs treating serious or life-threatening illnesses and provide sponsors with similar advantages, with BTD regulated by the Center for Drug Evaluation and Research (CDER) and RMAT by the Center for Biologics Evaluation and Research (CBER). In 2021, CBER approved three therapeutics with the RMAT designation pathway and CDER approved 14 through BTD [20, 21].

In 2018, the FDA’s Oncology Center of Excellence (OCE) initiated Real-Time Oncology Review (RTOR) to streamline the review process and further facilitate the availability of groundbreaking therapeutics to patients with life-threatening conditions [22, 23]. With RTOR, the sponsor and regulators carefully plan and agree on a strategy and timeline for the rolling submission of module components. Although the Prescription Drug User Fee Act (PDUFA) review clock does not officially begin until the last document is submitted, by supplying selected sections earlier, applications can sometimes be approved 3 to 4 months before the PDUFA goal date [24].

The European Medicines Agency (EMA) has also implemented several regulatory procedures designed to expedite patient access to new medicines, including Conditional Marketing Authorization (CMA), Exceptional Circumstances for rare diseases, Accelerated Assessment, Priority Medicines (PRIME) and Compassionate Use [25–29]. A common feature for eligibility for these pathways is an “unmet medical need.” Certain pathways like CMA also apply to orphan drug medicines which treat rare diseases (defined as those with a prevalence of 5 in 10,000 or less). However, the understanding of “unmet medical need” in the EU, where there is no formal definition, is very different from that laid out by the FDA [30]. Most of these pathways allow for the deferred submission of certain information as post-approval commitments such as phase III clinical data, long-term stability data, and other data to refine the product manufacturing control strategy. Each pathway follows its own timeframe and expedites the review and approval to varying extents. Of the available expedited approval pathways, only CMA and PRIME are potential options outside the scope of an emergency. In 2021, the EMA approved 13 drugs with CMA and six with PRIME designation [31].

During the COVID-19 public health emergency, the heightened need for vaccines and/or therapies resulted in the EMA allowing for a “rolling review” mechanism under the “EMA plan for emerging health threats” [32, 33]. With this review mechanism, the EMA and the sponsors preemptively agree to a series of rolling reviews with predefined document submission timelines. This allows documents to be submitted and reviewed as data become available. Once the application package is considered sufficiently complete, the sponsor can proceed to apply for CMA. As with other CMAs, additional data can be submitted post-approval. This process enabled a faster review of COVID-19 vaccines to meet the public health emergency.

Japan’s Pharmaceutical and Medical Device Agency (PMDA) has three main pathways available for expedited review of a marketing application (MA): priority review, conditional early approval, and Sakigake [34]. Priority review shortens the review time from 12 months to 9 months. The criteria for this pathway are that the drug treats a serious disease and/or shows clinical usefulness by meeting an unmet medical need or shows improved efficacy and safety compared to current options. Conditional early approval (CEA) applies to drugs where it is difficult to provide confirmatory trials or doing so would be too time consuming and the MA can be filed with adequate levels of efficacy and safety during exploratory trials. Confirmatory clinical trial data must be submitted post-approval. The Sakigake designation is aimed at drug products targeting unmet medical needs, or for the treatment of serious or life-threatening conditions, and pairs the sponsor with a PMDA staff review concierge to streamline communication throughout the development and application process. Sponsors also benefit from rolling reviews, a shortened review period, and the ability to provide submission materials in English. During the first 5 years, 37 drugs received this designation, and 10 were approved [35, 36].

Several international collaborative review initiatives across regulatory agencies have been initiated to allow faster patient access to critical medicines, globally. Project Orbis and the Access Consortium are two examples of collaboration amongst regulators that have been operating for several years. Project Orbis was initiated in May 2019 by the FDA’s OCE to leverage the scientific and regulatory knowledge across participating countries to enable faster global access to crucial cancer treatments [22, 37]. Review efficiency is improved through resource sharing (e.g., by sharing reviews between the participating regulatory agencies). As of August 2022, eight countries take part in Project Orbis: Australia, Brazil, Canada, Israel, Singapore, Switzerland, the United Kingdom (UK) and the US. In 2021, the FDA approved 26 marketing applications supported by Project Orbis [38]. The Access Consortium, established in 2007, is a collaborative effort between regulatory agencies in Australia, Canada, Singapore, Switzerland, and the UK, that would benefit from work-sharing, enhancing synergy, and increased efficiency by reducing duplication [39, 40]. In addition, sponsors have the advantage of receiving consolidated questions from multiple markets, predictable timelines for information requests (IR), and potentially, near-simultaneous approvals in multiple markets.

The International Coalition of Medicines Regulatory Authorities (ICMRA) is a voluntary organization comprised of 24 member regulatory agencies and 15 associate member agencies. Together, they work to identify areas of potential synergies and leverage existing resources whenever possible [41]. At the start of the COVID-19 pandemic, the ICMRA issued a statement of collaboration. This led to a large regulator-industry workshop in July 2021 which was well-attended and had broad outreach [42, 43]. From these discussions, the framework for two collaborative pilot programs was developed: one for facility inspections, and the other for the assessment of CMC post-approval changes. The two pilot programs began accepting applications in June 2022 and are both under oversight by the ICMRA working group for pharmaceutical quality knowledge management systems [39, 44]. The primary goal is to explore the feasibility of collaboration for facility inspections and increased harmonization of data expectations for CMC post-approval submissions, both efforts that can be supported by improved managing of source data.

## CMC challenges in regulatory submissions of products under accelerated development

**Fig. 1:**
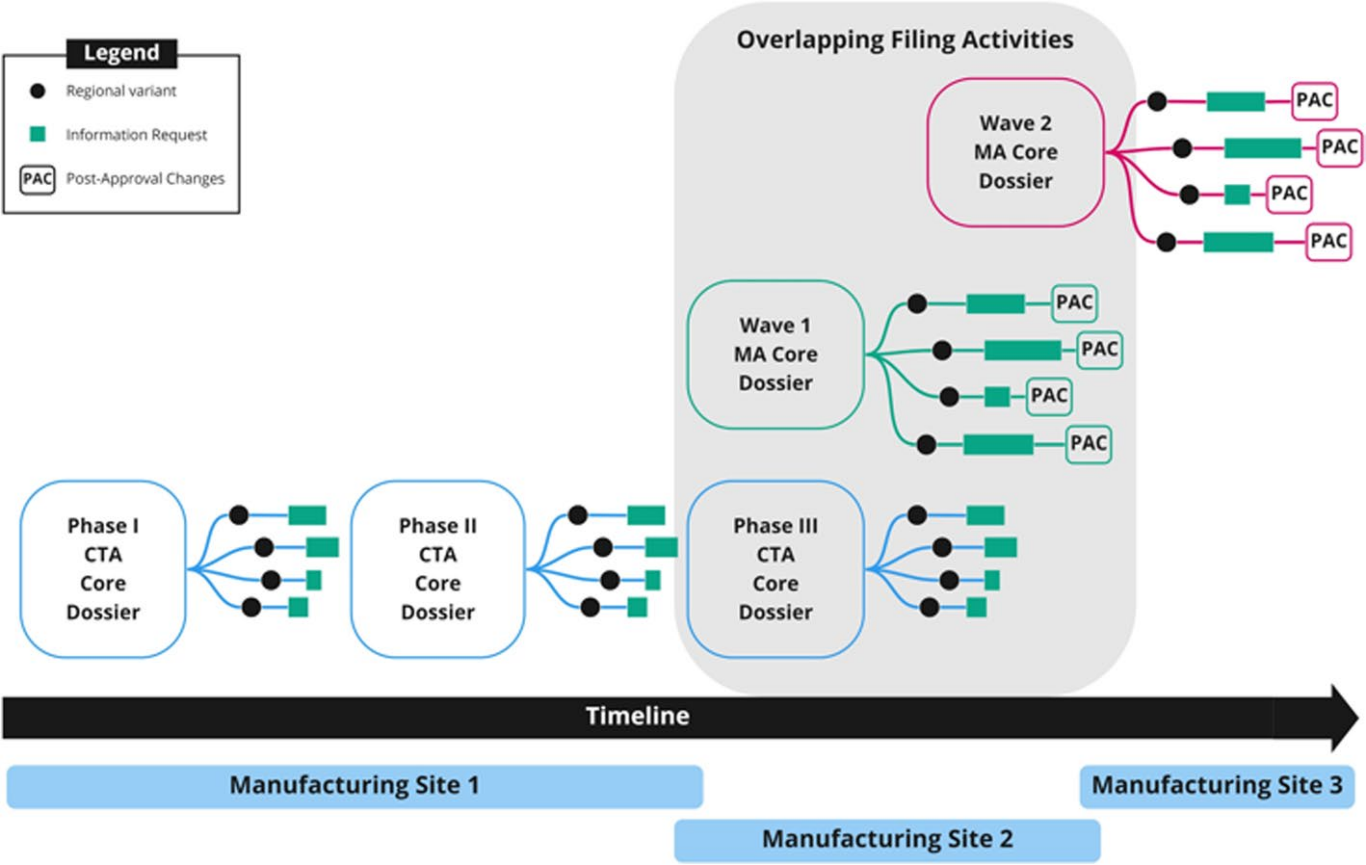
Compounding submission volume and complexity during development. The quantity of dossiers, country variants, information requests, and manufacturing changes substantially increases over time. For accelerated programs, later stages of development are often characterized by overlapping milestones and filing expectations, wherein the phase 3 trial may overlap with the filing of the marketing application. The marketing application may be filed in multiple discrete “waves” including groupings of different regions, wherein authoring, review, and approval timelines may overlap

Based on a benefit-risk assessment, many accelerated procedures outlined in the previous section allow phase 2 data that shows clear indication of efficacy to be used as the pivotal clinical data for submission with phase 3 confirmatory studies to be started during the MA review cycle for completion post-approval [45]. However, accelerated clinical development poses major product development challenges by compressing essential CMC activities into shortened clinical timelines, thereby potentially placing the required CMC data on the critical path for marketing authorization submission [4–6, 46]. Optimal dose selection is often pending at the time of first subject dosing in critical registrational trials. This forces the development of multiple presentations at risk and may require additional clinical stage amendments to support the selected presentation intended for commercialization. Furthermore, the transition to commercialization may include changes to the manufacturing site and/or scale to meet projected demand for the product, which in part explains why data to support process validation and product stability are on the critical path for the target submission date. These effects of acceleration can result in limited commercial product supply at time of product launch onto the market, increasing the complexity of the CMC strategy and the number of post-approval changes, and thereby increased regulatory submissions. Clearly, management of accelerated global filings is complex and requires careful tracking of various data requirements which increase as a product progresses through development, expands to include submissions in multiple countries, and manages responses to health authority questions along with post-approval changes and commitments (Fig. 1). This is a highly resource-intensive process that can be protracted for some years and is further delayed by reliance on archaic filing mechanics to relay CMC data. Therefore, efficiency gains in the processes that govern a filing strategy are of critical importance. These complexities in managing the submission planning can be addressed in part by robust yet flexible knowledge management which may be afforded by SCDM that works directly with content and data rather than documents. Ultimately, in the current non-digital document-based submission paradigm, incorporation of critical CMC data into the filing requires significant time and resources for authoring, data verification, formatting, and publishing, all of which negatively compete with the expedited clinical timeline. To minimize the timeframe for preparation of CMC information, innovative approaches and tools are needed that facilitate efficient management of CMC data in regulatory submissions.

Health authorities and industry pharmaceutical trade organizations have recently discussed tools to support CMC acceleration. Along with existing submission strategies such as rolling reviews and negotiations on uses of the established term “prior knowledge,” several new ideas were introduced and considered during the COVID-19 emergency and may potentially be carried over to expedite product development outside of emergency circumstances. Under PRIME, the EMA released a valuable toolbox for CMC acceleration that was available in a draft form through 2021 and was published in 2022 [47]. Likewise, FDA released a new Manual of Policies and Procedures (MAPP) titled “Quality Assessment for Products in Expedited Programs” and plans to start the CMC Development and Readiness Pilot (CDRP) in 2023 to accelerate CMC development for Investigational New Drug (IND) sponsors at both CBER and CDER [48–50].

In addition to the regulatory tools and strategic methodologies described above, utilization of technology to streamline the data management, authoring, and verification of CMC information presents a comprehensive approach for alleviating the impact of present acceleration challenges. Capabilities like SCDM provide enhanced functionality in comparison to conventional submission preparation processes, aiming to improve efficiency while simultaneously promoting a science and risk-based assessment by reducing the time between digital data generation and data availability in the filing for health authority review [46]. SCDM could also support optimization of reviewing procedures for health authorities through sharing of information across applications or through a cloud-based system. Above all, the value of SCDM would be most evident for sponsors simultaneously managing multiple expedited submissions worldwide, with responses to health authority questions and subsequent post-approval variations needed to optimize the manufacturing process over time [4–6]. The ability to repurpose prior knowledge and other product filing data libraries rapidly for numerous submissions would provide an enormous gain in efficiency [47, 51–53].

## Current applications of SCDM in the pharmaceutical industry

**Fig. 2:**
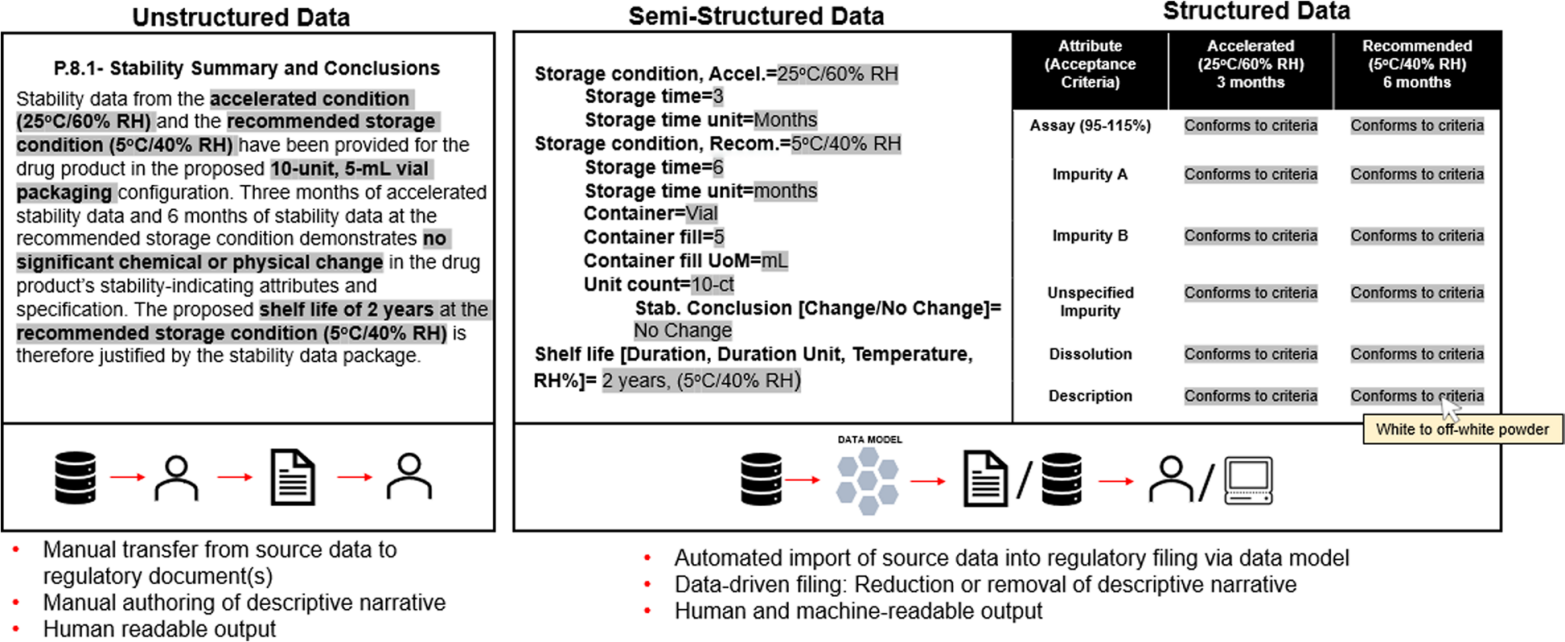
Data formatting conventions for regulatory information. Sample stability data illustrating unstructured, semi-structured, and structured data formats are depicted. Key content and data input fields are highlighted in gray for visualization. While unstructured data characterizes much of the current format, SCDM principles are optimally applied to semi-structured or structured data. While data appearing in a structured table appears simplified, the raw data is still accessible via a variety of viewing formats. While not inclusive of all possible interfaces and navigation options, an example shown here utilizes “hover text” that shows the corresponding data value when the “conforms to criteria” field is highlighted

In addition to targeted CMC acceleration tools and expedited regulatory pathways, Information Technology (IT) infrastructure can be effectively leveraged to manage resource demands and complex filing scenarios for industry and global health authorities. While individual organizations may pursue a multi-pronged approach to IT modernization that consists of various elements of Pharma 4.0, such as AI and machine learning, SCDM is of particular importance for enhancing data management capabilities to support regulatory filings. SCDM is a component-based approach to information management that can drive regulatory modernization and provide solutions for efficiency challenges faced by sponsors and regulators. SCDM can incorporate structured and semi-structured data, wherein structured data is largely tabular and in a highly ordered sequence which typically ascribes to a controlled terminology list. Semi-structured data retains a partial hierarchal format but allows for flexibility through input of free text. Conversely, unstructured data is free text that contains terms that provide essential information, typically in paragraph format (Fig. 2).

**Fig. 3:**
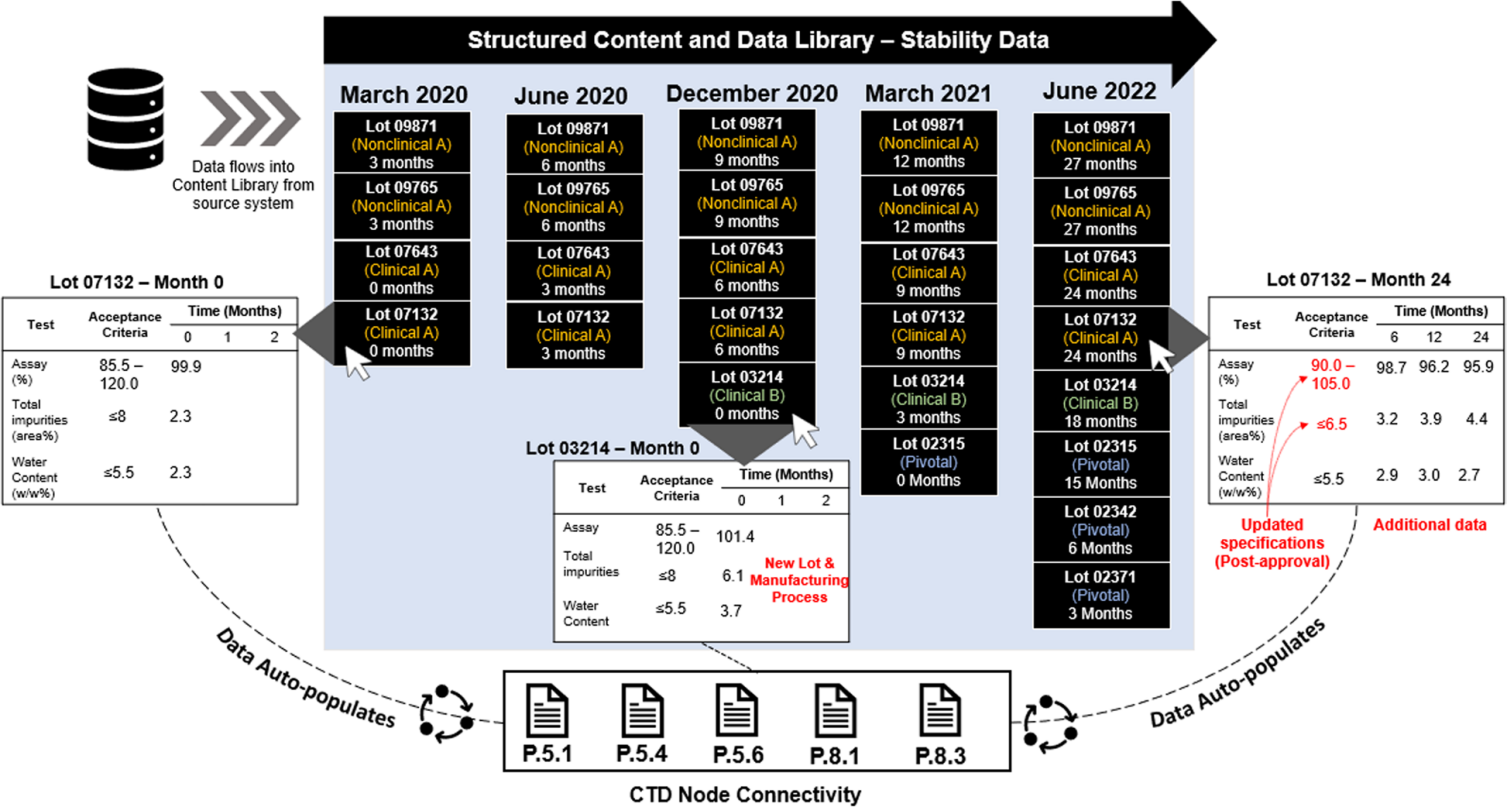
Structured content and data library for stability data. An overview of a structured content and data library housing stability information is depicted. Each black box constitutes a “content block” that is attributed to a specific lot. Inside each content block, information such as test method, acceptance criteria, and timepoints are accessible. Data is imported into the library directly from the data source (ex. LIMS, eLN). Over time, as development progresses, stability data continually accumulates across lots and, additionally, new lots are developed as the manufacturing process is optimized. Content blocks data elements are updated in real time as new data points become available from data sources. Content blocks and data elements can be “pulled” from the library to auto-populate module 3 CTD sections to prepare for submission

SCDM is based on the concept of a centralized and interoperable data repository or library that is comprised of modular content authoring blocks and data elements that build upon each other to create a product-specific record. Using this concept, a content block can be defined as a container for a stack of data elements, wherein the data elements relate to one another and commonly appear together to comprise a data set or description of a specific attribute (Fig. 3). SCDM leverages the reusability of content from a given content block to build related content blocks or to facilitate reuse of the same content block across applications, thereby drastically limiting the need for consecutive authoring, reviewing, and approval cycles of the same content block or data element [7]. Data elements or content blocks can be distributed across multiple electronic common technical document (eCTD) sections for various regulatory filing requirements and can be efficiently updated throughout the product lifecycle. This interoperability can ease the burden of repetitive authoring and allow for the auto-population of content, which can be updated in real-time as changes or new data are applied. The narrative can be refreshed accordingly with author input as necessary. Eliminating data verification alone with the use of a validated SCDM system significantly reduces review time and resources, as there is no manual data transcription. As module 3 of the eCTD is comprised of designated datasets and referenced details that are repeated across multiple nodes (or CTD sections) in an unstructured format, regulatory dossiers become increasingly cumbersome to maintain and continually update, particularly in the post-approval environment wherein there can be multiple variations or supplements in preparation simultaneously. With SCDM, the individual content blocks can be structured, mapped to different eCTD nodes where they are needed, and linked to a centralized data element repository for storage and maintenance, enabling an easier authoring process and enhancing automation [12]. Instead of editing, data verifying, and republishing a full document, modifications can be made to individual components as needed, which will then auto-update in each section in which the components appear. In this way, changes can be applied across multiple sections and multiple regions simultaneously. From a technical perspective, SCDM can facilitate parallel review activities across different agencies by helping to manage multiple country-specific variations that impact the same CTD section, such as specification sections, which may be different across regions due to individual agency regulatory requirements and preferences. An additional compelling advantage to having such a centralized data repository is to support the acquisition and integration of a program/company, where the new sponsor company is responsible for retrieving archived data to support new submissions at various points in the product lifecycle, often in the absence of the technical development experts.

In addition to supporting efficiency by optimizing how content is authored, managed, and reused, SCDM can also enable digitalization by decoupling CMC data from discrete documents, to move towards a data-driven approach. As discussed previously, structured content using a re-usable structured content and data library can house modular blocks of information (data with metadata tags) to be used internally to build the content of an on-demand internal data report, as well as externally to address a health agency data request [12, 54]. For example, in FDA submissions, a drug master file (DMF) is a current mechanism for reusing data, which can be replaced with SCDM for future applications. This “data on demand” SCDM approach can be considered a technologically advanced version of the DMF mechanism. The DMF can be updated and maintained routinely in an automated manner independently from a given filing or application. For example, stability data stored in the cloud for different drug product CTDs can be directly pulled for a new submission using SCDM. In a similar manner as using data from the DMF, SCDM can share submissions and data in a digital format in the cloud. Easy digital access to data used in amendments, variations, annual reports, and supplements can be repurposed in specific areas of a submission for use globally depending on agency requests [55].

Similarly, SCDM can bring data from multiple management systems together and allow for data interconnectivity across systems through integrations. Currently, data are stored and managed across multiple internal systems, such as Laboratory Information Management Systems (LIMS), Electronic Lab Notebooks (eLN), Product Lifecycle Management (PLM) software, and company data lakes. This makes it difficult to transfer, assimilate, and track source data in preparation for regulatory submissions. SCDM can be leveraged to build connectivity between systems by structuring and contextualizing content. The structured content and data library can ultimately be used in combination with an enterprise data lake (EDL) to achieve a unified CMC data model that establishes semantic context between data elements and is fully interoperable across systems and applications [12].

The use of SCDM in a regulatory and pharmaceutical context is in its relative infancy with comparatively little real-world experience versus traditional operating models for submission assembly. Biopharmaceutical companies, as well as global health authorities, have begun to preliminarily explore SCDM-based architecture and solutions internally within recent years as part of a larger shift towards regulatory modernization and digital system expansion, but it is not yet a widespread industry standard or regulatory expectation [7]. Several other industries with their own sets of complex regulations have demonstrated successful use cases digitization, including banking and financial services, the food and beverage industry, automotive industry, marketing, and aerospace, which showcases the suitability of structured content across complex, regulated environments [56–61].

There is increasing support towards implementing structured content-based solutions in life sciences and pharmaceutical organizations which is demonstrated, in part, by the emergence of commercial tools offered by software and data management support vendors for SCDM solutions tailored for biopharmaceutical use cases. Over the past 5 years, as companies have adopted SCDM approaches internally, technological solution developers have evolved alongside the industry, as many vendors now showcase customer testimonials and successful case studies from across multiple domains of biopharmaceutical companies, including research and development, regulatory, labeling, and operations. Select examples of platform-based applications developed to support the management of CMC regulatory data include Docuvera’s^™^ CMC solution, which provides a single-source authoring system for reviewing, approving, publishing, reusing, and managing CMC content; Cognition’s^®^ Lighthouse^™^ system, which can generate automated structured stability data reports from data imported manually or from source systems of record; and QbDVision^®^, which has a suite of content management solutions for organizing and reusing CMC data throughout the product lifecycle [62–64]. Other solution providers, such as Cognizant^®^, IQVIA^®^, OpenText^™^, Esko, InteliNotion, and Vasont ^®^, have created SCDM tools specifically for life science and biopharmaceutical information management, which can be applied across domains, including CMC [56–59]. Vendors such as Veeva and LORENZ are creating specific toolsets to help organize structured data in line with upcoming regulatory data requirements [65, 66]. Additionally, companies may develop in-house applications using commercially available data visualization programming packages (e.g., R Shiny) that are specifically customized to integrate with existing business data architecture and deliver structured content solutions [67]. The availability of configurable commercial software and Software as a Service (SaaS) platform solutions increases the accessibility of SCDM for wider implementation across biopharmaceutical companies with differing organizational sizes, technological capabilities, and data complexity.

## Emerging regulatory enablers of SCDM

While adoption of SCDM solutions can support efficiency and optimization of resource management, particularly in accelerated filing scenarios, there is presently no requirement, or significant external impetus, for biopharmaceutical developers to pursue these advancements in technology as dossiers can be compiled and organized manually using current operating procedures. However, as the industry collectively advances towards digital maturity as part of Pharma 4.0, the need to reconfigure from unstructured to structured data management solutions will be further escalated by key imminent developments that are gaining momentum globally with several health authorities.

### Emerging submission requirements for structured, standardized CMC and quality data

Data standardization is a key driver for automation and represents an important step towards structuring data by enabling consistency and interoperability. Presently, there are no data standardization requirements for CMC data that are submitted to regulatory authorities. While sponsors must meet the requirements outlined in local legislation and are advised to follow relevant International Council for Harmonization of Technical Requirements for Pharmaceuticals for Human Use (ICH) guidance and regional and country-level guidance, there are no data standards currently in use that govern how data are presented, formatted, and structured in the regulatory dossier. Although ICH M4Q guidelines provide baseline content and structural requirements, they lack the level of section-by-section detail and granularity that would be needed to support a fully structured application. The lack of data standardization leads to the increasing heterogeneity of submissions across sponsors and regulators, as sponsors may submit data in multiple formats while regulators at different agencies may use different tools and metrics for assessment. Differences in syntax and nomenclature can make it difficult to map pieces of information that are conceptually identical. For example, the pharmaceutical dosage form categories “capsule, hard” and “hard capsule” are definitionally the same and ontologically should be modeled as the same object (thing). Similarly, a release specification result for a critical quality attribute may be operationally identical to the stability time zero result for that attribute and should be modeled for both purposes. However, if this mapping is not properly conducted or completed, these items could be rendered as separate objects and create limitations for machine-based assessment and content auto-population. Ultimately, these differences in terminology across regions create unneeded complexity that contributes to variations in data interpretation.

The International Organization for Standardization (ISO) originally published the Identification of Medicinal Product (IDMP) standards in 2012 with the objective of enabling simplicity and consistency of medicinal product and substance data that are exchanged between sponsors and regulators [68]. IDMP standards span multiple different domains in the pharmaceutical industry in a limited scope, including product labeling, safety, pharmacovigilance, and CMC, focusing on the elements that are deemed most essential for medicinal product and substance identification. Specific coverage areas include product and substance nomenclature, manufacturers, characteristics, marketing authorization status, and packaging details. Several regulators, including the EMA, have communicated their intentions to require compliance with ISO IDMP standards, but formal guidance towards implementation is pending across multiple regions.

Specifically, the EMA plans to implement ISO/IDMP compliance by utilizing an approach based on the four domains of data within pharmaceutical regulatory processes including Substance, Product, Organization and Referentials (SPOR). EMA’s efforts intend to facilitate the reliable exchange of key medicinal product information in a structured, standardized, consistent, and efficient manner in line with the healthcare industry [69]. While the implementation timeline for SPOR is to be determined, the EMA is expected to initiate the implementation of IDMP standards through the related Digital Application Dataset Integration (DADI) project, which will replace the current PDF-based electronic Application Form (eAF) with a web-enabled form [70].

Separate from SPOR, the FDA has developed an alternative data standardization initiative, focusing specifically on CMC data requirements under the Pharmaceutical Quality/Chemistry, Manufacturing, and Controls (PQ/CMC) project that expands the IDMP concept across CMC. The initiative aims to enable sponsors to pivot toward providing structured data applications to improve interoperability between stakeholders and increase the efficiency of the FDA’s review of CMC data. As part of the 2012 *Food and Drug Administration Safety and Innovation Act* (FDASIA), the FDA released an initial Federal Register Notice in 2017 on a series of structured data elements intended to provide structure across a variety of CTD module 3 sections, including stability, specification, analytical methods, and batch analyses [71]. In 2022, the FDA shared a refined and expanded version of the PQ/CMC data elements including mapping to Health Level 7 (HL7) Fast Healthcare Interoperability Resources (FHIR) [72, 73].

The FDA’s PQ/CMC standards as well as the EMA’s SPOR rely on exchange specifications to enable the transfer of information from sponsors to health authorities. Both standards utilize FHIR as an exchange standard. HL7 FHIR is an open-source data format with an accompanying application programming interface (API) that leverages flexible and modifiable resource elements to bring structure and standardization to healthcare information while allowing for targeted customization [74]. Through FHIR, sponsors and health authorities will be able to securely exchange electronic correspondence through FHIR messages, which can include a bundle of structured information that is downloadable in a variety of formats including XML and JSON. FHIR can accommodate regional variations in terminology lists, which supports its use as a flexible, global solution for standardization [75–79].

In addition to FDA’s and EMA’s efforts to develop FHIR-compliant data standards, a new HL7 FHIR project was established in late 2022 with the goal of developing global internal data standards to enable exchange of data across biopharmaceutical industry data systems. These standards are being developed in collaboration with industry stakeholders and will maintain alignment with both PQ/CMC and ISO IDMP to the extent feasible. The project, entitled, “Data Exchange Industry – Pharmaceutical Quality (dx-PQ)” will cover a variety of scenarios in its initial iteration, including technology transfers, manufacturing process changes, and stability data updates [80].

### Adoption of data analytics tools to support regulatory review

In 2018, the FDA first shared its plans to create a risk-based, computer-aided reviewing tool entitled Knowledge-Aided Assessment and Structured Application (KASA) [81]. The KASA tool would input received structured data into an analytical database that utilizes risk assessment algorithms to evaluate key CMC and quality attributes, such as manufacturing facilities, pharmaceutical development, and control strategy [82]. The KASA tool is intended to address inefficiencies in the review process, promote consistency and objectivity during analysis, and keep pace with technological innovation in line with the goals of the Pharmaceutical Quality for the 21st Century Initiative [76]. The FDA’s related PQ/CMC initiative would support implementation of KASA by enabling sponsors to provide structured data in an appropriate format at the time of regulatory submission, which could then be directly imported into the KASA tool for analysis. Both initiatives represent a change in thinking towards an objective data-driven, risk-based decision-making approach wherein unstructured narrative is minimized in favor of usable, assessment-ready data. The initial scope to pilot the KASA tool included Abbreviated New Drug Applications (ANDA) for generic drugs, as this represents both a high-volume area for FDA reviewers and a high-impact area for consumers. The solution is anticipated to evolve over the next 5 years to include a review of New Drug Applications (NDAs), Biologics License Applications (BLAs), and post-approval changes [76–79].

### Innovations in cloud-based technology

Cloud-based computing harnesses the collective power of numerous interconnected servers to enable a customizable suite of computing and processing capabilities, including analytical tools, software applications, remote data storage, and access to volumes of information on-demand via the intranet/internet. Flexibility, scalability, and interoperability are among the key value propositions for cloud services, which allow systems within the cloud-based ecosystem to communicate with one another, bringing data from different sources together to achieve new levels of accessibility. For pharmaceutical companies and regulators, the practicality of SCDM can be further augmented by a cloud-enabled unified data model that connects data to allow for the seamless access and use of data [12].

Regulators are increasingly moving towards cloud-based systems to optimize processes such as data exchange, assessment, and archiving. In the US, as part of the FDA’s Technology Modernization Action Plan (TMAP), the agency is exploring next steps and requirements for migrating the FDA’s IT infrastructure to a cloud-enabled model. As reported in the 2022 TMAP Anniversary Report, 35% of the agency’s systems are utilizing cloud-based approaches, which has contributed to significant resource savings and assisted with the development of scalable next-generation data centers [83]. Similarly, in the EU, the EMA has shared its intent to migrate its IT systems entirely to the cloud by 2025 to allow the agency to further modernize and digitize its operations [84]. To date, many of the externally facing systems used by the EMA have already adopted a cloud-enabled infrastructure, such as the EMA’s IRIS platform, which can be utilized to initiate a number of regulatory procedures such as applying for orphan drug designation, seeking scientific advice, and providing updates on marketing authorization status for products [44, 85].

While it is important for regulators to upgrade and modernize their own internal systems to promote continuous technological innovation and advancement, regulators and sponsor companies would benefit from having a common, international, cloud-based information exchange platform to conduct correspondence and initiate regulatory procedures. In 2020, Accumulus Synergy, a nonprofit organization and technical solution innovator, was formed in response to this growing need. Accumulus Synergy is creating a cloud-enabled solution that fosters collaboration between and across regulators and industry, enables real-time regulatory submission, and allows for dynamic structured data exchange for CMC and other domains [86]. With support from 12 biopharmaceutical sponsors, Accumulus is developing a scalable, democratized platform with shared and secure workspaces that will be made broadly accessible by all life science companies and health authorities around the globe. The long-term vision of the platform is to decrease regulatory submission and review timelines, as well as development costs, to accelerate availability of medicines for patients. As an integral aspect of the vision to establish a cloud-based data exchange platform, the Accumulus platform aims to support and aid adoption of the aforementioned CMC data standards currently in development through a flexible and accessible user interface and structured data repository. Notably, Accumulus Synergy is a primary stakeholder in the development of the HL7 industry internal CMC data standard, Data Exchange Industry – Pharmaceutical Quality (dx-PQ) [80].

## Applying the principles of SCDM through the product lifecycle regulatory process: a case study

**Fig. 4:**
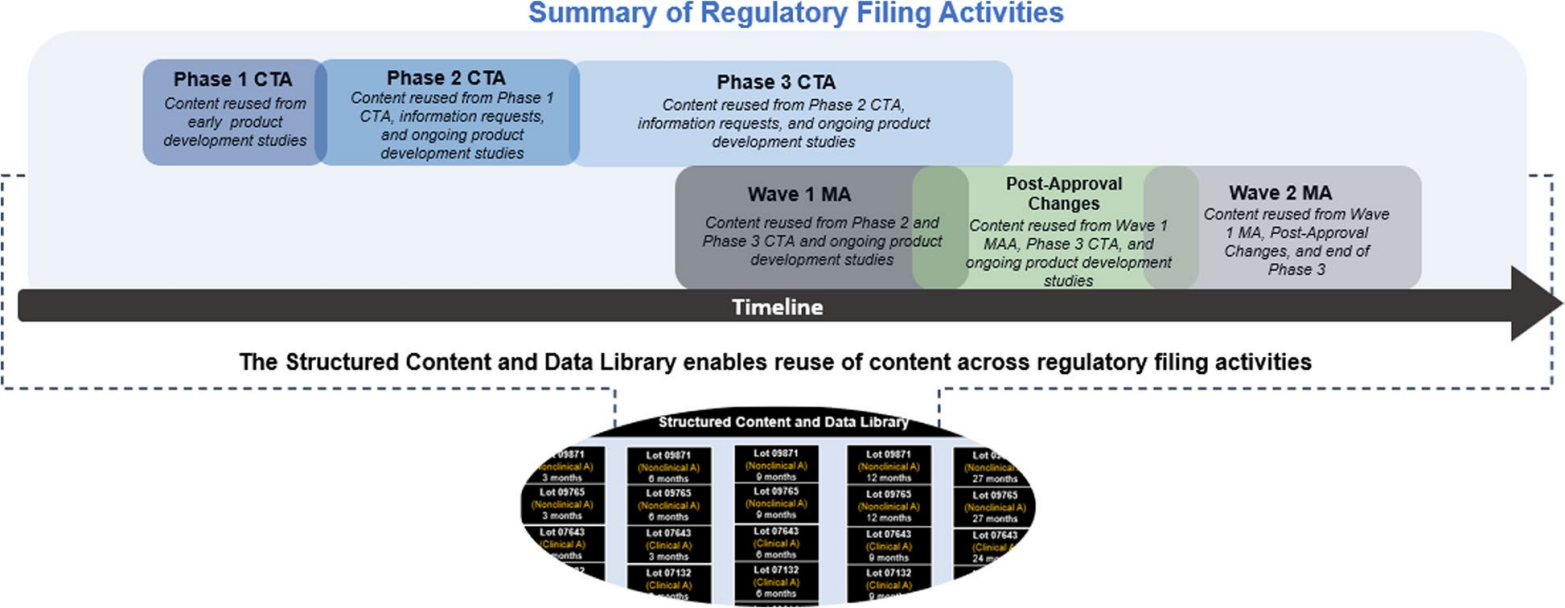
Case study summary timeline. The timeline outlines the major regulatory filing milestones for the hypothetical case study. Each filing activity milestone is able to leverage substantial content and data reuse supported by the structured content and data library to streamline authoring and submission

In this section, a hypothetical case study is presented as an example of how SCDM can be leveraged to increase efficiency in CMC submissions for a medicinal product in an accelerated development scenario. Figure 4 illustrates the high-level development timeline demonstrating this example product. Early MA submissions (“wave 1”) are planned on the basis of compelling phase 2 clinical data. As a result, the clinical trial application (CTA) submissions supporting confirmatory phase 3 studies may be ongoing at the same time, resulting in overlapping workload and adding to project management challenges. The timing of necessary changes to the manufacturing process, product formulation, strength, and container closure system or site must be carefully considered in order to ensure uninterrupted supply to patients globally. Due to the compressed timelines, certain elements of product optimization as well as many necessary changes may need to be deferred to the post-approval stage, at the same time as filing of the “wave 2” MAs.

The product is identified in pre-clinical development as a potential target for undergoing accelerated development. Leveraging SCDM technologies, discovery, and preclinical data are appropriately logged and categorized in a cloud-based EDL and structured content and data library where data are centrally stored, organized, and made accessible for future component content use. Before first-in-human (FIH) studies can take place, the regulatory team initiates the process of drafting CTAs to support initial studies, focusing on inaugural target markets. The team accesses the data content from pre-clinical studies in the EDL and structured content library to map relevant component content blocks to each respective CTA filing. This process enables a direct digital connection between material used in preclinical and early clinical studies, which has historically been challenging as it spans many years and personnel changes. Continual data collection from these early process development experiments is enabled by tagging and mapping information that can populate product and region-specific content blocks in a data library. These content blocks contain all the CMC information that can be built sequentially as the product development program matures, as shown in Fig. 3. Following any required updates due to agency questions from the initial CTA applications, content blocks can be re-used for streamlined file-building and submission operations in subsequent CTA filings, in line with the global regulatory strategy. As opposed to authoring documents specific to each filing, the product data are accessible and reusable via a multi-dimensional data network of semantically connected data elements throughout the product lifecycle. In lieu of tracking down and aligning documents with specific phases of development and regions, data elements of the content record can be flexibly deployed to the required filings from a core content repository. This agility is especially valuable given the shifting timelines that are often associated with accelerated development.

As clinical studies progress, alignment between the clinical and CMC activities must be continually evaluated and maintained, as changes to clinical development milestones may substantially impact CMC resource allocation and timelines. For example, if late-breaking clinical results were to suggest that the product delivers substantial benefit in a subset of the patient population with a specific genetic mutation, development of a companion diagnostic would be required which might cause delays to the overall program timeline. SCDM better supports incorporation of any CMC updates that are needed during this delay since these are captured within the content library via content blocks and data elements. These elements can be readily deployed to relevant parts of the regulatory applications concurrently for all countries. The advantage of SCDM in this scenario is agility through rapid incorporation of updated CMC information in submissions, in response to clinical timeline changes as they occur in real-time.

When compelling phase 2 clinical results are received, applications are made to gain expedited regulatory pathway designations (BTD in the US, PRIME designation in the EU, and Sakigake in Japan). Further complexities are evident if an expedited review designation is received in one market and not in others, resulting in staggered submission planning and constantly shifting scenarios. Unanticipated early approvals also may necessitate acceleration of scale-up and site transfer activities to meet ongoing clinical trial supply demand and commercial requirements for each of the multiple countries targeted for wave 1 MA submissions. The team is also simultaneously managing CTA submissions for multiple confirmatory phase 3 clinical trials to support additional data collection across regions. As shown in Fig. 1, this represents an intense period of overlapping submissions and responses to health authority requests for information for both CTAs and MAs. Participation in collaborative regulatory efforts such as Project Orbis requires further coordination and handling of multiple simultaneous requests for information, often with short turnaround times as well as country-specific CMC summaries such as quality assessment aids required by regulators.

**Fig. 5:**
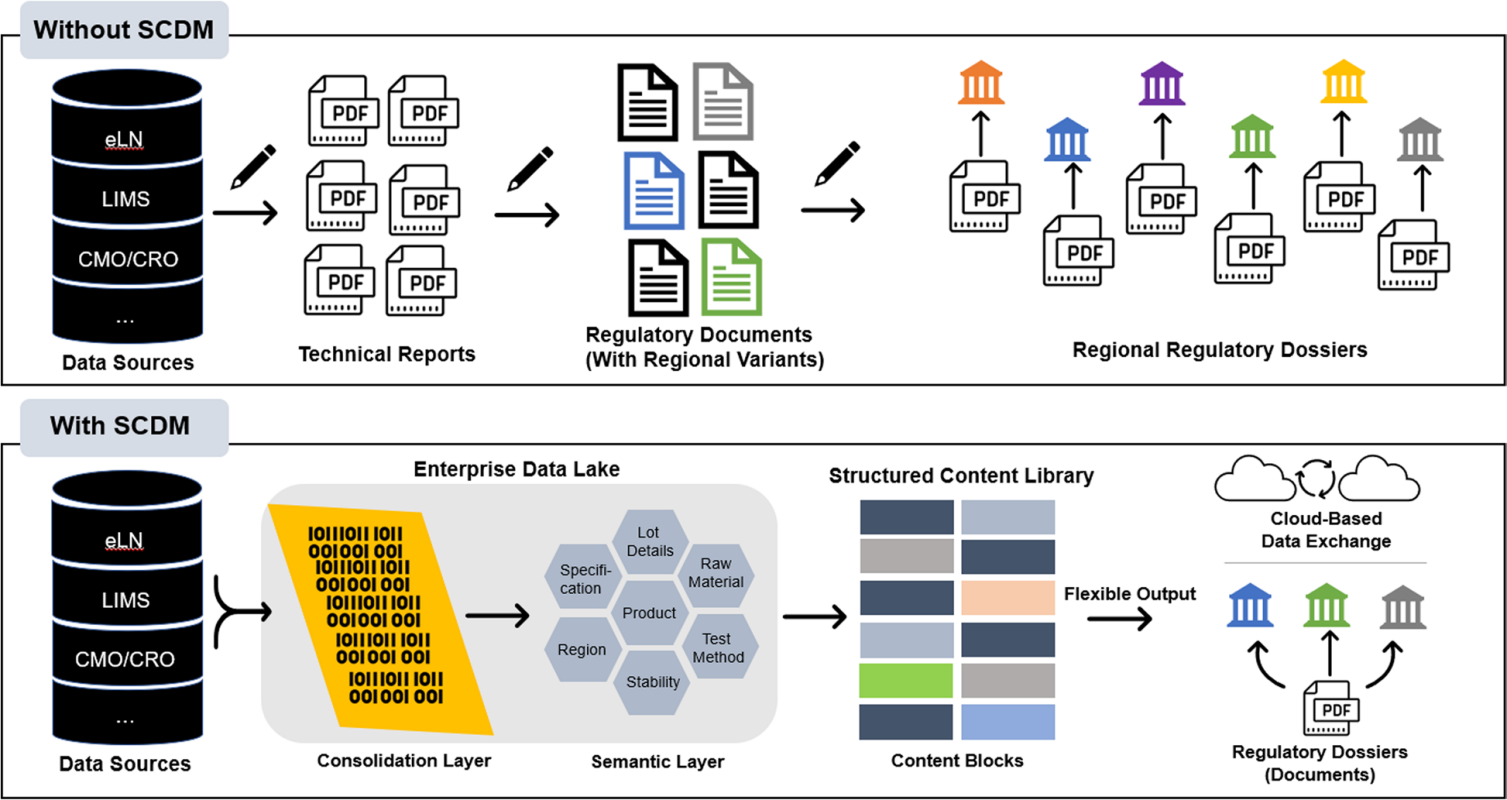
Comparison of regulatory submission processes with and without SCDM. A comparison of regulatory CMC submission assembly and dispatch is shown. Without SCDM, data is manually transcribed from the source into a technical report. The technical report is then used as a source document for a regulatory document, of which there are multiple regional variants created manually. Compilations of documents are then sent individually to health authorities for review, requiring the company to maintain multiple regional dossier variants. With SCDM, manual authoring steps can be reduced or eliminated as content and data can be taken directly from the source system, processed and semantically mapped via an enterprise data lake, and made available for incorporation in a regulatory filing via the structured content and data library, which is made up of content blocks. The content blocks can be reorganized, updated, and customized according to regulatory objectives and regional filing needs. The structured content and data library can achieve flexible output options, including electronic data submission, cloud-based information transfer, and printable paper-based filings

Without SCDM, the MAs would be prepared following current practices, in which a core CMC dossier is first created from data sources with aggregate information requirements for all countries. From this global dossier, the team would need to prepare multiple country-specific MAs and ongoing CTAs. Since this team does have an established SCDM workflow, they do not need to manually generate the different documents and variants. The tagged data in the content block is retrieved from the EDL and structured content library and loaded into the relevant sections of the application. Thus, data can travel from a centralized EDL that is comprised of a consolidation layer (that accrues relevant product data from various data sources) and a semantic layer (that connects the product data elements based on an ontological data model which defines the relationships, classifications, and associations of data elements in a cloud-based ecosystem) (Fig. 5).

Most of the health authorities in this hypothetical case study accept MA submissions in eCTD format, but some have the appropriate IT infrastructure to accept data in FHIR exchange format. SCDM tools enable the same datasets to be reused across a variety of outputs and file types, including paper, PDF, and FHIR-based data submissions. SCDM ultimately provides a hierarchy of inherent structure defined by semantic relationships between pieces of data which can be represented across file formats and presentations. This flexibility is a key aspect of SCDM’s utilization as it can accommodate varying levels of technological maturity across global regulators to simultaneously produce traditional (paper, PDF) and data-driven submissions. SCDM would additionally streamline the MA process by ensuring that country-specific summaries required by regulators can be completed accurately and expeditiously.

As the team is preparing the initial MAs for submission, advancements in process development and manufacturing are occurring simultaneously. As mentioned earlier, scale-ups and site transfers may be needed to meet commercial and clinical supply chain requirements, resulting in complex tracking of these changes across MAs and CTAs. The team must decide which changes to include in the MAs or in post-approval filings. The team files its MA first in one jurisdiction; At this time, two manufacturing sites A and B are included but plans for a third site C are underway to meet projected demand. By the time the team files the MA in additional jurisdictions, the final wave 1 countries, all sites are operational and thus included in the MA. The team can leverage SCDM to update the regional MAs in real-time based on manufacturing site readiness and data availability.

After the wave 1 MA submissions, several hundred information requests (IRs) are received from multiple agencies. It is advantageous for the sponsor to have traceable country-specific data to quickly and accurately provide a response to the agency requests given the time constraints for IRs imposed by the various agency regulatory frameworks. With SCDM, all this information is readily available, as data on stability, comparability, and number of lots manufactured is mapped to specific applications and regions for which they were included. Without SCDM, it would be difficult to track what information was included for which country.

The use of prior knowledge and SCDM can provide structured content blocks to leverage related stability data from similar products for justification in rapid decision making. Since the timeline is accelerated, limited stability data are available from lots that can be justified as primary stability lots per ICH definitions [87]. The team leverages prior knowledge from representative products to refine the shelf-life analysis and model the stability profile for the candidate product. Additionally, the use of SCDM enables updates to stability data simultaneously in different submissions as they become available. At the time of the initial MA filing, the team has 12 months of stability data for two lots and 9 months for the third lot. A shelf-life at approval of at least 18 months is proposed, and this generally requires real time data from at least 3 representative lots to be provided during review. The filing team queries through the product-specific records and identifies two commercial products with similar manufacturing processes, formulations, and structural characteristics, but with slightly different SKUs and packaging configurations. One of the commercial products has 18 months of stability data while the other has 36 months. The team provides these stability data to supplement and model the predicted expiry supporting justification for a reasonable shelf-life at original approval. With the use of SCDM, the product team can supply these data to aid the approval process and are later able to update filings in real-time once product-specific testing is completed. 

After the product is approved, it becomes necessary to carefully manage the post-approval changes in each country such as the addition of a new manufacturing site. As shown in Fig. 4, the team is preparing wave 2 MAs while also submitting manufacturing site C information to many of the wave 1 countries as a post-approval change. SCDM can help by centrally storing all the site-specific information through data elements that can then be picked up and utilized for any one of the submissions.

Lastly, by using SCDM, the product team more effectively manages a post-approval change to the product presentation in all countries. The product is initially approved as a once-weekly subcutaneous injection that is transported in vials and administered for 6 months. The once-weekly course of administration is burdensome for patients who must arrange transportation to their healthcare provider’s office. This burden negatively impacts adherence to the dosing regimen. After wave 1 MA approvals, the sponsor optimizes the delivery with a new administration regimen which requires one injection every month and is shipped as a pre-filled syringe. To this end, MAs for wave 2 countries will contain this presentation mode but wave 1 MAs will need to be updated according to each country’s reporting requirements. SCDM helps the team to manage which submissions are required for specific countries and updates the information needed. This results in a more seamless rollout of the new presentation in pre-filled syringes which improves patient adherence and outcome.

Ultimately, throughout the MA filing process, authoring and submission timelines could be reduced by as much as 50% using SCDM in comparison to the traditional process. Additional savings can be realized by enabling faster filings in subsequent markets, thus reducing initial MA approval times in wave 2 regions to 4-5 years as compared to a current average of 7-10 years.

## The evolution of CMC submissions

**Fig. 6:**
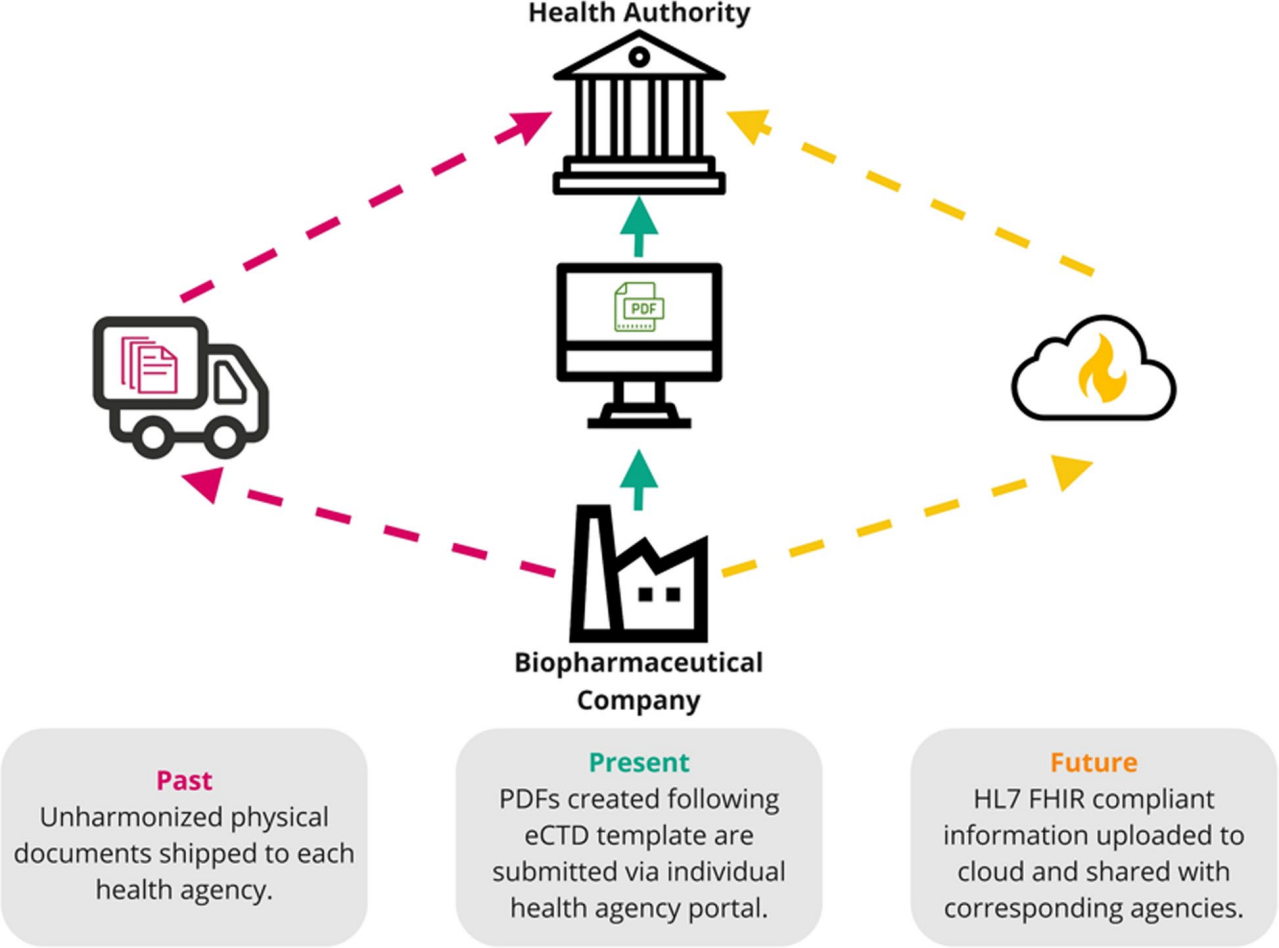
Summary of past, present, and future regulatory filing processes. While physical, paper-based submissions were the standard operating model in the past, the current regulatory framework in many major markets supports electronic submission of regulatory information in eCTD format. The conversion to an electronic system has ushered in multiple benefits, but there are ways to improve. In the future, electronic filings can achieve standardization and interoperability across data systems via HL7 FHIR, which is adapted for a real-time, cloud-based exchange ecosystem

Until the creation of the ICH CTD, physical copies of regulatory applications with unharmonized formatting were shipped on pallets to major market agencies as illustrated schematically in Fig. 6. The physical logistics and paper reviews in preparations for shipping the application pallets were a massive hurdle for companies in an increasingly globalized marketplace. The CTD as a harmonized work product was a valuable advancement that created a structure for regulatory submissions that would be recognized by ICH member jurisdictions [88]. However, the medium by which information was shared continued to be a physical paper copy. As technology evolved, the eCTD emerged moving a physical paper copy into an electronic structure and format. The ICH eCTD was first adopted in 2003 by the EMA [89]. This transition from physical to electronic paper has made it easier for regulatory agencies to review the submissions by providing the ability to hyperlink to different locations within the structure of the eCTD. Sponsors benefited from reduced allocation of resources to the preparation, storage, and shipping of physical documents. The process was a favorable and noteworthy transition as the industry entered the twenty-first century, providing value to sponsors and health authorities by harmonizing the global regulatory submission process. The industry and health authorities can provide significant benefit to patients by accelerating the logistics of regulatory submissions and the review processes through the use of digital technologies and adoption of an easily searchable and verifiable data-centric approach that would be superior to the document-based eCTD.

The next milestone will be a move to cloud-based submission systems with the use of targeted SCDM components. Directing structured, verified source data components into regulatory submissions will enable transcendence beyond the eCTD. The cloud-based structure will contain “open” shared and “closed” restricted areas with portals to control access within and between these areas. This will promote concurrent and collaborative regulatory submissions and reviews between the sponsor, agencies, and third-party collaborators, with a focus on reporting of data as opposed to document creation. This future state requires sponsor and regulatory agency endorsed collaborative efforts equivalent to the global developments of the CTD and eCTD. Data-centered submissions can potentially advance inter-agency product discussions and provide CMC improved surveillance methods by accessing Module 3 data on demand for agency-related questions and thus drive more efficient regulatory oversight.

As the biopharmaceutical industry gravitates towards patient centricity, both sponsors and regulators must continue to work not only to protect the safety of the patient population but must also focus on delivering and maintaining supply of therapies to patients in a more timely and efficient manner. Numerous industries have already leveraged twenty-first century technologies to enhance speed and productivity [12]. However, the biopharmaceutical industry is struggling to determine how best to use current technologies in a highly regulated environment [12]. Today, regulatory success for the biopharmaceutical sector remains solely dependent on the exchange of electronic or physical documents, limiting communication with health authorities to “in series” transactions as illustrated in Fig. 1.

Health authority initiatives like KASA, PQ/CMC, ISO/IDMP (SPOR), and ICH M4Q(R2) have the opportunity to lead the way toward digitalization and globally aligned structured regulatory filings [77, 90–92]. Although these initiatives are in their early stages, they are providing the vital foundation for a modernized regulatory filing system. Both PQ/CMC and ISO/IDMP (SPOR) initiatives are FHIR-based and provide a common global language for sponsors and health authorities to leverage for the secure exchange of electronic information. The alignment of the described health authority initiatives with FHIR data exchange and a cloud-based ecosystem would revolutionize regulatory filings, transcend the current capabilities of the ICH eCTD, and move the biopharmaceutical industry into a modern application architecture.

Despite its many demonstrated advantages, there are some challenges related to SCDM implementation and use of cloud-based platforms that revolve around data localization, management, access, privacy, and legality [93]. Regulatory agencies want to preserve their autonomy and companies need to ensure their intellectual property (IP) is safeguarded. Decisions as to whether data will be centralized and managed by a third party, or a hybrid method will need to be made. Standards will need to be put in place regarding system interoperability and cybersecurity to protect patient and IP data from breaches. Application programming interface (API) specifications will need to be structured such that each player in the regulatory ecosystem is only sharing what is necessary and internal information is secure and protected. Moreover, the systems will need to be built with global compatibility in consideration. Region-specific legal hurdles will also need to be identified before implementation. Legal considerations regarding dataset server localization, submission archival, and electronic verifications will need to be addressed.

Another challenge that hinders SCDM implementation is that the current system requires further standardization and structuring. At present, long, unstructured narratives are commonly included in the CMC sections of the eCTD. Although these are intended to be data-driven sections, companies may share more information and supplementary analysis than is necessary, often motivated by a desire to avoid future inquiries. Discussions will need to be had to provide clear standardized guidelines on what is and is not essential in the regulatory decision-making process. Such conversations can be supported by regulators’ interest in developing risk-based review algorithms, such as KASA [79]. Shorter, auto-generated summaries can be used to replace extensive narratives where applicable. This will allow for more effective resource allocation to the reviewing of essential data as opposed to reviewing extraneous text creating a more efficient and nimble submission and review process.

In the future, AI pattern recognition could be harnessed for strategic planning as well as to track patient safety. AI-based technology is being adopted for pharmacovigilance to help with adverse event intake processing and could potentially be used to assist with the decision-making process for safety correlations [94, 95]. Additionally, AI would be useful in searching through data to identify patterns in agency requests for further information, establish precedent in regulatory decision making, and help seek consistency in agency reviews [96]. AI in regulatory may begin streamlining work by aiding in supporting efficient data collection and therefore improving regulatory discussion and collaboration through natural language processing and vision applied to regulatory intelligence. As an example, AI could be harnessed to address responses to agency questions by drafting a response from content within the data lake and based on previous responses. At the post-approval stage, AI implementation can be used in the industry to learn continuously about each product change and more accurately identify higher and lower change-associated risks to better match regulatory reporting requirements including documentation. AI could learn from similar agency requests or questions made previously for other products, evaluate similarity between those products, and use those prior knowledge data to assess the risk presented by a given change. Understanding of risk could potentially lower the reporting category for that change. AI can be used together with other tools to provide essential information to a health authority including data and modeling to support the risk assessment and to mitigate existing risks. From a regulator’s perspective, AI can enable iterative learning about a particular change relative to other changes related to a product or different products or across sponsors. Health authorities would also be able to take each sponsor’s distinct quality systems into account. SCDM would then be able to facilitate the gathering of all available knowledge to assess the risk of a particular change more appropriately in a systematic manner and thus evaluate the critical aspects of its impact.

## Conclusion

Adoption and utilization of expedited regulatory review pathways are increasing on a global scale. While this supports the acceleration of product availability for patients with unmet medical needs, there are many efficiency and resource challenges that must be mitigated in accelerated development environments. Navigating the myriad regulatory requirements and timelines requires strategic coordination.

It is possible to accelerate drug development and advance treatments to patients faster if product data, and data from applicable prior knowledge from other molecules, is managed more efficiently through a SCDM approach. The current system locks data for each product into PDF documents which are manually generated, verified, reviewed, published, and submitted. Health authorities must in turn manually retrieve submitted data locked in PDF documents to be analyzed and compiled into their systems. This is a laborious, inefficient, and error-prone process. Sourcing prior knowledge data from separate product dossiers is a particular challenge for a document-based system. Employing SCDM would mean that structured data blocks can be deployed to relevant sections of the eCTD and could even be employed across different product eCTD sections. This process would ensure data integrity and traceability, making it more amenable to the use of tools like modeling. A stepwise approach to implementing SCDM in regulatory submissions can be envisioned.

Sponsors, regulators, and patients all share the same goal: faster access to safe and effective treatments. Towards this common goal, sponsors, health authorities, and third-party organizations need to work together to develop the infrastructure and supportive regulatory policies needed to enable the utilization of information technology to accelerate the preparation, submission, and review of regulatory filings. The vision for an efficient and expedited regulatory submissions process is the use of SCDM in single-sourced filings and collaborative agency reviews, with manual submission tasks replaced by auto-populated filings, assisted by system interoperability and API integration in a cloud-based exchange platform. A cloud-based submission system with SCDM integration which allows sponsors to reuse information without the need to generate PDF documents will provide a more seamless submission and lifecycle management process. Health authorities will be empowered to make better decisions, based on data that is more traceable, reliable, and transparent. CMC data is used, updated, and reused hundreds if not thousands of times throughout a product’s lifecycle. Improved management of these data and its usability can propel therapeutic innovation into the future. It is up to sponsors and regulators to work together and build this new infrastructure that enables acceleration by eliminating underlying inefficiencies. It is imperative that patient treatment should not be delayed because of outdated regulatory document preparation and submission systems.

## Abbreviations

API: Application programming interface
AI: Artificial intelligence
ANDA: Abbreviated New Drug Application
BTD: Breakthrough therapy designation
CBER: Center for Biologics Evaluation and Research
CDER: Center for Drug Evaluation and Research
CMC: Chemistry, Manufacturing, and Controls
CDRP: Chemistry, Manufacturing, and Controls Development and Readiness Pilot
CTA: Clinical trial application
CTD: Common technical document
CEA: Conditional early approval
CMA: Conditional marketing authorization
DADI: Digital Application Dataset Integration
eAF: Electronic application form
eCTD: Electronic common technical document
ELN: Electronic Lab Notebooks
EMA: European Medicines Agency
FHIR: Fast Healthcare Interoperability Resources
FIH: First in human
FDA: Food and Drug Administration
FDASIA: Food and Drug Administration Safety and Innovation Act
HL7: Health Level 7
IDMP: Identification of Medicinal Product
IR: Information request
IT: Information technology
ICMRA: International Coalition of Medicines Regulatory Authorities
ICH: International Council for Harmonization of Technical Requirements for Pharmaceuticals for Human Use
ISO: International Organization for Standardization
IND: Investigational new drug
KASA: Knowledge-Aided Assessment and Structured Application
LIMS: Laboratory Information Management Systems
MAPP: Manual of Policies and Procedures
MA: Marketing application
NDA: New Drug Application
OCE: Oncology Center of Excellence
OMS: Organisation Management Services
PQ/CMC: Pharmaceutical Quality/Chemistry, Manufacturing, and Controls
PDUFA: Prescription Drug User Fee Act
PRIME: Priority Medicines
PLM: Product Lifecycle Management
PMDA: Pharmaceuticals and Medical Devices Agency
PMS: Product Management Services
RTOR: Real-time Oncology Review
RMAT: Regenerative Medicine Advanced Therapy
SaaS: Software as a Service
SOP: Standard Operating Procedure
SPOR: Substance, Product, Organization and Referentials
TMAP: Technology Modernization Action Plan
UK: United Kingdom
US: United States

## References

[1] Berdel WE (2021). Unintended regulatory caused early death-a difficult endpoint in cancer patient care and treatment. Cancers (Basel).

[2] Friedersdorf C (2021) *The death toll of delay.* The Atlantic. Available from: https://www.theatlantic.com/ideas/archive/2021/08/fda-delays-carry-death-toll/619871/

[3] Helwick C (2015) *Delays in drug approval are deadly, highlighting the need for improved regulatory efficiency.* The ASCO Post. Available from: https://ascopost.com/issues/october-25-2015/delays-in-drug-approval-are-deadly-highlighting-the-need-for-improved-regulatory-efficiency/

[4] Potter CJ, Yuan H, Cauchon NS, Chang LL, Blaettler D, Kin DW, Millili PG, Mazzola G, Ocheltree T, Tyler SM, Taber G, Watson TJ (2019) *Accelerated Pharmaceutical product development, registration, commercialization, and life cycle CMC lessons, Part 1*. Pharmaceutical Engineering. Available from: https://ispe.org/pharmaceutical-engineering/ispeak/accelerated-pharma-product-development-registration

[5] Potter CJ, Yuan H, Cauchon NS, Chang LL, Blaettler D, Kin DW, Millili PG, Mazzola G, Ocheltree T, Tyler SM, Taber G, Watson TJN (2019) *Accelerated pharmaceutical: product development, registration, commercialization & life cycle CMC lessons, Part 2*. Pharmaceutical Engineering. Available from: https://ispe.org/pharmaceutical-engineering/accelerated-pharmaceutical-product-development-registration

[6] Dye III, Groskoph ESJG, Kelley B, Millili GP, Nasr MM, Potter CJ, Thostesen ES, Vermeersch H (2015) *CMC Considerations when a Drug Development Project is Assigned Breakthrough Therapy Status* Pharmaceutical Engineering. Available from: https://ispe.org/pharmaceutical-engineering/january-february-2015/cmc-considerations-when-drug-development-project#:~:text=CMC%20Considerations%20when%20a%20Drug%20Development%20Project%20is%20Assigned%20Breakthrough%20Therapy%20Status,-Earl%20S.&text=Assignment%20of%20Breakthrough%20Therapy%20(BT,a%20%E2%80%9Cconventional%E2%80%9D%20development%20program

[7] Algorri M, Cauchon NS, Abernathy MJ (2020) Transitioning chemistry, manufacturing, and controls content with a structured data management solution: streamlining regulatory submissions. J Pharm Sci 109(4):1427–1438 10.1016/j.xphs.2020.01.020 32004537

[8] Gardner N (2021) *Data integrity and pharma 4.0*. Available from: https://www.thermofisher.com/blog/connectedlab/data-integrity-and-pharma-4-0/?ce=E.22CMD.DS109 .06571.01&cid=E.22CMD.DS109.06571.01&iq=IQLAAKGACZFALKMAXR&ef_id=CjwKCAjw1ICZBhAzEiwAFfvFhHai57BUPOSeo3z7VRyDsgbU6Pdbm7yrVFn_C2Csbn86fzPaCwhjARoCUvsQAvD_BwE:G:s&s_kwcid=AL!3652!3!563674789876!p!!g!!pharma%204.0&gclid=CjwKCAjw1ICZBhAzEiwAFfvFhHai57BUPOSeo3z7VRyDsgbU6Pdbm7yrVFn_C2Csbn86fzPaCwhjARoCUvsQAvD_BwE. Accessed 10 Jan 2023

[9] Reinhardt IC, Oliveira J, Ring D (2023) *Industry 4.0 & the future of the pharmaceutical industry*. Pharmaceutical Engineering. Available from: https://ispe.org/pharmaceutical-engineering/march-april-2021/industry-40-future-pharmaceutical-industry. Accessed 10

[10] International Society of Pharmaceutial Engineering (ISPE). *Pharma 4.0™* Available from: https://ispe.org/initiatives/pharma-4.0. Accessed 13 Sept 2022

[11] QbDVision. (2020) *ICH, QbD, Pharma 4.0: One and the Same*. Available from: https://www.qbdvision.com/ich-qbd-pharma/. Accessed 22 Aug 2022

[12] Ahluwalia K, Abernathy MJ, Beierle J, Cauchon NS, Cronin D, Gaiki S, Lennard A, Mady P, McGorry M, Sugrue-Richards K, Xue G (2021) The future of CMC regulatory submissions: streamlining activities using structured content and data management. J Pharm Sci 111(5):1232–1244 10.1016/j.xphs.2021.09.046 34610323

[13] Helfand C (2019) *If pharma looks slow to adopt AI, it’s got good reason, expert says*. FIERCE Pharma. Available from: https://www.fiercepharma.com/marketing/if-pharma-looks-slow-to-adopt-ai-there-s-good-reason-expert. Accessed 12 Jan 2023

[14] Manzano T, Canals A (2022) *Measuring Pharma’s Adoption of Industry 4.0*. Pharmaceutical Engineering. Available from: https://ispe.org/pharmaceutical-engineering/january-february-2022/measuring-pharmas-adoption-industry-40

[15] Hwang TJ, Darrow JJ, Kesselheim AS (2017) The FDA’s expedited programs and clinical development times for novel therapeutics, 2012–2016. JAMA 318(21):2137–2138 10.1001/jama.2017.14896 PMC5820715 29209711

[16] Friends of Cancer Research (2023) *Modernizing expedited development programs*. Friends of Cancer Research Annual Meeting 2020. Available from: https://friendsofcancerresearch.org/wp-content/uploads/Modernizing_Expedited_Development_Programs-2020_0.pdf. Accessed 12

[17] Beierle J, Cauchon NS, Graul TW, Hedberg Y, Holm MB, Lepore JV, MacKenzie R, Mistry K, Qian X, Robinson K, Rullo G, Tang KT, Watson T (2022) *Toward a *single global control strategy: industry study Pharmaceutical Engineering. Available from: https://ispe.org/pharmaceutical-engineering/january-february-2022/toward-single-global-control-strategy-industry

[18] FDA (1988) Available from: https://archives.federalregister.gov/issue_slice/1988/10/21/41492-41527.pdf#page=25. Accessed 13 Jan 2023

[19] FDA. Guidance for Industry Expedited Programs for Serious Conditions - Drugs and Biologics (2014) Available from: https://www.fda.gov/media/86377/download. Accessed 22 Aug 2022

[20] FDA. *Advancing Health Through Innovation: New Drug Therapy Approvals CDER 2021* Available from: https://www.fda.gov/media/155227/download. Accessed 20 Aug 2022

[21] FDA. CBER Regenerative Medicine Advanced Therapy (RMAT) Approvals (2022) Available from: https://www.fda.gov/vaccines-blood-biologics/cellular-gene-therapy-products/cber-regenerative-medicine-advanced-therapy-rmat-approvals. Accessed 25 Aug 2022

[22] Algorri M, Acharya A, Bernstein J, Cauchon NS, Chen XH, Huynh-Ba K, Krantz C, Tao Li YL 6, McLamore S, Roberts SW, Schwinke D, Shah R, Schirmer A, Strickland H, Tang K, Watson T (2022) 19 *Meeting Report: Advancing Accelerated Regulatory Review with Real-Time-Oncology Review (RTOR), Project Orbis, and the Product Quality Assessment Aid* AAPS Open 8 Available from: https://aapsopen.springeropen.com/articles/10.1186/s41120-022-00066-1 10.1186/s41120-022-00066-1 PMC9734574 36530577

[23] FDA. Real-time review of drug applications is now a reality (2018) 18 Sept. Available from: https://www.fda.gov/drugs/real-time-review-drug-applications-now-reality-september-20-2018-issue. Accessed 22 Aug 2022

[24] Feng C, Virparia R, Mui ET (2021) Analysis of the real-time oncology review (RTOR) pilot program for approvals of new molecular entities. Ther Innov Regul Sci 55(4):881–888 10.1007/s43441-021-00296-7 PMC8081281 33913098

[25] EMA. *Conditional marketing authorization*. Available from: https://www.ema.europa.eu/en/human-regulatory/marketing-authorisation/conditional-marketing-authorisation. Accessed 29 Aug 2022

[26] EMA. *Orphan designation: marketing authorization*. Available from: https://www.ema.europa.eu/en/human-regulatory/marketing-authorisation/orphan-designation-marketing-authorisation. Accessed 29 Aug 2022

[27] EMA. *Accelerated assessment*. Available from: https://www.ema.europa.eu/en/human-regulatory/marketing-authorisation/accelerated-assessment. Accessed 29 Aug 2022

[28] EMA. *PRIME: priority medicines*. Available from: https://www.ema.europa.eu/en/human-regulatory/research-development/prime-priority-medicines. Accessed 29 Aug 2022

[29] EMA. *Compassionate use*. Available from: https://www.ema.europa.eu/en/human-regulatory/research-development/compassionate-use. Accessed 29 Aug 2022

[30] Vreman RA, Heikkinen I, Schuurman A, Sapede C, Garcia JL, Hedberg N, Athanasiou D, Grueger J, Leufkens HGM, Goettsch WG (2019). Unmet Medical need: an introduction to definitions and stakeholder perceptions. Value in Health.

[31] EMA. Annual Report (2021) Available from: https://www.ema.europa.eu/en/documents/annual-report/2021-annual-report-european-medicines-agency_en.pdf. Accessed 13 Jan 2022

[32] EMA (2022) EMA initiatives for acceleration of development support and evaluation procedures for COVID-19 treatments and vaccines. Available from: https://www.ema.europa.eu/en/documents/other/ema-initiatives-acceleration-development-support-evaluation-procedures-covid-19-treatments-vaccines_en.pdf. Accessed 13 Jan 2023

[33] Marinus R, Mofid S, Mpandzou M, Kühler TC (2022). Rolling reviews during COVID-19: the European Union experience in a global context. Clin Ther.

[34] PMDA (2021) Expedited regulatory pathways in Japan. Available from: https://www.youtube.com/watch?v=P6z3MGDhYh4. Accessed 13 Jan 2023

[35] Tanaka M, Idei M, Sakaguchi H, Kato R, Sato D, Sawanobori K, Kawarasaki S, Hata T, Yoshizaki A, Nakamura M, Ikuma M (2021) Achievements and challenges of the Sakigake designation system in Japan. Br J Clin Pharmacol 87(10):4027–4035 10.1111/bcp.14807 33694268

[36] MHLW (2020) Notification: PSEHB/PED No. 0831/6: handling of designation of pioneer drugs

[37] De Claro RA, Spillman D, Hotaki LT, Shum M, Mouawad LS, Santos GML, Robinson K, Hunt M, Healy C, Chan A, Looi YH, Rodrigues C, Rohr UP, Walther C, Pazdur R (2020). Project Orbis: Global Collaborative Review Program Clin Cancer Res.

[38] FDA. Project Orbis (2022) Available from: https://www.fda.gov/about-fda/oncology-center-excellence/project-orbis. Accessed 29 Aug 2022

[39] ICMRA. *Pharmaceutical quality - regulatory collaboration pilots: call for industry applications* Available from: https://icmra.info/drupal/strategicinitatives/pqkms/pq_pilots_call_for_industry_applications. Accessed 29 Aug 2022

[40] ICMRA (2022) A regulatory pharmaceutical quality knowledge management system (PQ KMS) to enhance the availability of quality medicines. Available from: https://www.icmra.info/drupal/strategicinitatives/pqkms/joint_reflection_paper. Accessed 16 Sept 2022

[41] ICMRA. International Coalition of Medicines Regulatory Authorities (2022) Available from: https://icmra.info/drupal/en. Accessed 29 Aug 2022

[42] ICMRA. ICMRA-industry virtual workshop report on enabling manufacturing capacity in the COVID-19 pandemic (2021) Available from: https://www.icmra.info/drupal/sites/default/files/2021-10/covid-19_manufacturing_capacity_ws_report.pdf. Accessed 10 Jan 2023

[43] *An Industry proposal: recommendations to support the rapid increase of manufacturing capacity for the production of COVID-19 therapeutics and vaccines*. ICMRA workshop on enabling manufacturing capacity in the COVID-19 pandemic 2021 28 June Available from: https://www.ifpma.org/wp-content/uploads/2021/07/Industry_Proposal_Mfg_Capacity_COVID-19-v18Feb2021_revJune2021.pdf. Accessed 22

[44] EMA. *Welcome to IRIS*. Available from: https://iris.ema.europa.eu/. Accessed 02 Sept 2022

[45] Nakajima EC, Drezner N, Li X, Mishra-Kalyani PS, Liu Y, Zhao H, Bi Y, Liu J, Rahman A, Wearne E, Ojofeitimi I, Hotaki LT, Spillman D, Pazdur R, Beaver JA, Singh H (2022) FDA approval summary: sotorasib for KRAS G12C-mutated metastatic NSCLC. Clin Cancer Res 28(8):1482–1486 10.1158/1078-0432.CCR-21-3074 PMC9012672 34903582

[46] Popkin ME, Goese M, Wilkinson D, Finnie S, Flanagan T, Campa C, Clinch A, Teasdale A, Lennard A, Cook G, Mohan G, Osborne MD (2022). Chemistry manufacturing and controls development, industry reflections on manufacture and supply of pandemic therapies and vaccines. AAPS J..

[47] EMA. Toolbox guidance on scientific elements and regulatory tools to support quality packages for PRIME and certain marketing authorisation applications targeting an unmet medical need (2022) Available from: https://www.ema.europa.eu/en/documents/scientific-guideline/toolbox-guidance-scientific-elements-regulatory-tools-support-quality-data-packages-prime-certain_en.pdf

[48] FDA. PDUFA reauthorization performance goals and procedures fiscal year 2023 through 2027 (2022) Available from: https://www.fda.gov/media/151712/download. Accessed 10 Aug 2022

[49] Food and Drug Administration Federal Register. Quality Management Maturity for Finished Dosage Forms Pilot Program for Domestic Drug Product Manufacturers; Program Announcement (2020) Available from: https://www.federalregister.gov/documents/2020/10/16/2020-22976/quality-management-maturity-for-finished-dosage-forms-pilot-program-for-domestic-drug-product. Accessed 10 Dec 2022

[50] FDA. Manual of Policies and Procedures MAPP 5015.13 “Quality assessment for products in expedited programs” (2022) Available from: https://www.fda.gov/media/162786/download. Accessed 9 Jan 2023

[51] EMA. Meeting Report: Joint BWP/QWP workshop with stakeholders in relation to prior knowledge and its use in regulatory applications (2018) Available from: https://www.ema.europa.eu/en/documents/report/meeting-report-joint-biologics-working-party/quality-working-party-workshop-stakeholders-relation-prior-knowledge-its-use-regulatory-applications_en.pdf

[52] EMA. Toolbox guidance on scientific elements and regulatory tools to support quality data packages for PRIME and certain marketing authorisation applications targeting an unmet medical need (2022) 22 April 2022. Available from: https://www.ema.europa.eu/en/documents/scientific-guideline/toolbox-guidance-scientific-elements-regulatory-tools-support-quality-data-packages-prime-certain_en.pdf

[53] EMA. Meeting Report: Workshop with stakeholders on support to quality development in early access approaches (i.e. PRIME, Breakthrough Therapies) (2018) Available from: https://www.ema.europa.eu/en/documents/report/report-workshop-stakeholders-support-quality-development-early-access-approaches-ie-prime_en.pdf

[54] Macdonald JC, Isom DC, Evans DD, Page KJ (2021) Digital innovation in medicinal product regulatory submission, review, and approvals to create a dynamic regulatory ecosystem-are we ready for a revolution? Front Med (Lausanne) 8:660808 10.3389/fmed.2021.660808 PMC8183468 34109196

[55] IBM. *What is a data fabric?* Available from: https://www.ibm.com/topics/data-fabric. Accessed 14 Sept 2022

[56] ArborSys. *Structured content management solutions*. Available from: http://www.arborsys.com/structured-content-management-solutions.html. Accessed 30 Aug 2022

[57] DitaExchange. *Structured content management for financial services*. Available from: https://ditaexchange.com/financial-services/. Accessed 30 Aug 2022

[58] ESKO. *Customer case studies*. Available from: https://www.esko.com/en/brands/case-studies. Accessed 30 Aug 2022

[59] OpenText™. *Read customer success stories*. Available from: https://www.opentext.com/customers. Accessed 30 Aug 2022

[60] Richardson M (2022) *Data management matters*. Aerospace Manufacturing 2020 23 November 2020. Available from: https://www.aero-mag.com/data-management-matters/. Accessed 30

[61] Studer S *Component content management use cases*. Zia Consulting 2020 22 Apr 2020. Available from: https://www.ziaconsulting.com/component-content-management/use-cases/. Accessed 14 Aug 2022

[62] Cognition (2022) *Why pharmaceutical companies look to lighthouse to generate electronic reports for CMC-related submissions* Available from: https://fs.hubspotusercontent00.net/hubfs/250507/Why%20Pharmaceutical%20Companies%20Look%20to%20Lighthouse%20to%20Generate%20Electronic%20Reports%20for%20CMC-related%20Submissions_Drug%20Stability_Cognition%20Corporation_January%202022.pdf. Accessed 14 July 2022

[63] Docuvera *Efficiently create and update CMC documentation throughout the entire pharmaceutical manufacturing process*. Available from: https://docuvera.com/chemical-manufacturing-and-controls/. Accessed 30 Aug 2022 2022

[64] QbDVision. *Discover all your knowledge can do* Available from: https://www.qbdvision.com/structured-platform/

[65] Veeva *IDMP Readiness Center*. Available from: https://www.veeva.com/products/vault-platform/vault-rim-idmp-resource-hub/

[66] Lorenz *Product information and lifecycle management with LORENZ drugTrack*. Available from: https://www.lorenz.cc/Solutions/idmp/

[67] GitHub IDG (2022) *Automate your workflow from idea to production*. Available from: https://github.com/features/issues. Accessed November 29 2022

[68] Tranchard S (2017) *Revised IDMP standards to improve description of medicinal products worldwide*. ISO. Available from: https://www.iso.org/news/ref2234.html

[69] EMA. *Substance, product, organisation and referential (SPOR) master data*. Available from: https://www.ema.europa.eu/en/human-regulatory/research-development/data-medicines-iso-idmp-standards/substance-product-organisation-referential-spor-master-data

[70] Miglierini G (2022) *The transition towards EMA’s new Digital Appliation Dataset Integration (DADI) user interface*. EIPG. Available from: https://eipg.eu/the-transition-towards-ema-new-digital-application-dataset-integration-dadi/

[71] Brennan Z (2017) *FDA looks to standardize PQ/CMC data and terminologies*. Regulatory Focus. Available from: https://www.raps.org/regulatory-focus%E2%84%A2/news-articles/2017/7/fda-looks-to-standardize-pq-cmc-data-and-terminologies

[72] FDA (2022) *Draft pharmaceutical quality/chemistry manufacturing and controls (PQ/CMC) data exchange*. Available from: https://www.fda.gov/media/157085/download. Accessed 16

[73] FDA (2022) Draft pharmaceutical quality chemistry manufacturing and controls (PQCMC) data exchange. SUPPORTING & RELATED MATERIAL. Available from: https://www.regulations.gov/document/FDA-2022-N-0297-0002. Accessed 17 Sept 2022

[74] HL7 (2022) Introducing HL7 FHIR. Available from: https://hl7.org/FHIR/summary.html. Accessed 30 Aug 2022

[75] HL7 (2022) *Code systems*. Available from: https://hl7.org/FHIR/terminologies-systems.html. Accessed 30

[76] Eglovitch JS (2021) *FDA taking incremental approach to launching KASA reviews*. Regulatory Focus. Available from: https://www.raps.org/news-and-articles/news-articles/2021/11/fda-taking-incremental-approach-to-launching-kasa

[77] IPQ. *FDA’s KASA and related PQ/CMC initiatives on improving CMC data structuring and sharing will help support ICH M4Q revision*. 2022 17 Feb 2022. Available from: https://ipq.org/fdas-kasa-and-related-pq-cmc-initiatives-on-improving-cmc-data-structuring-and-sharing-will-help-support-ich-m4q-revision/

[78] Kozlowski S, FDA/PQRI Conference on Advancing Product Quality 2021 *Knowledge-Aided and Structured Application (KASA) and Pharmaceutical Quality/CMC (PQ/CMC) Update:KASA for Biologics*. 5th. Available from: https://pqri.org/wp-content/uploads/2021/12/3-PQRI-KASA-for-Biologics_12-3-21_SKozlowski-FINAL-v1.pdf

[79] Roelofs B *What’s in a KASA? Knowledge-aided assessment and structured application (KASA) for biological products*. Well Characterized Biotechnology Products WCBP 2022. Available from: https://www.casss.org/docs/default-source/wcbp/2022-wcbp-speaker-presentations/speaker-presentations-roelofs-brian-cder-fda-2022.pdf?sfvrsn=bdc8a079_8

[80] HLA FHIR. Data exchange industry – pharmaceutical quality (dx-PQ) (2020) Available from: https://build.fhir.org/ig/HL7/uv-dx-pq/background.html

[81] Tarius (2017) *SAC Tracker Pharmaceutical Science and Clinical Pharmacology Advisory Committee*. Available from: http://www.sac-tracker.com/pscp-20180920-ba

[82] Yu LX, Raw A, Wu L, Capacci-Daniel C, Zhang Y, Rosencrance S (2019). FDA’s new pharmaceutical quality initiative: knowledge-aided assessment & structured applications. Int J Pharmaceutics: X.

[83] FDA. Modernization in Action 2022 (2022) Available from: https://www.fda.gov/files/about%20fda/published/Modernization_in_Action_2022.pdf

[84] EMA. European Medicines Agency Cloud Strategy (2022) Available from: https://www.ema.europa.eu/en/documents/other/european-medicines-agency-cloud-strategy-accelerating-innovation-digitalisation-better-public-animal_.pdf

[85] EMA. *Q&As on IRIS registration, login and RPI requests*. Available from: https://iris.ema.europa.eu/forums/whats-new/b9e932c2-3ad7-ea11-bf21-0003ff5fd63e. Accessed 02 Sept 2022

[86] Nogueira F (2022) *Welcome to Accumulus Synergy*. Available from: https://www.accumulus.org/. Accessed 9

[87] ICH. ICH Topic Q 1 A (R2) stability testing of new drug substances and products (2003) Available from: https://www.ema.europa.eu/en/documents/scientific-guideline/ich-q-1-r2-stability-testing-new-drug-substances-products-step-5_en.pdf

[88] ICH (2022) *History*. Available from: https://www.ich.org/page/history. Accessed 17

[89] Mezher M (2016) *Going digital: EMA to ditch paper, require electronic application forms* RAPS February 26, 2015. Available from: https://www.raps.org/regulatory-focus%E2%84%A2/news-articles/2015/2/going-digital-ema-to-ditch-paper,-require-electronic-application-forms

[90] EMA. Introduction to ISO identification of medicinal products, SPOR programme (2016) EMA/732656/2015. Available from: https://www.ema.europa.eu/en/documents/other/introduction-iso-identification-medicinal-products-spor-programme_en.pdf. Accessed 17 Jan. 2023

[91] FDA. *Pharmaceutical quality/chemistry, manufacturing & controls (PQ/CMC)*. Available from: https://www.fda.gov/industry/fda-data-standards-advisory-board/pharmaceutical-qualitychemistry-manufacturing-controls-pqcmc. Accessed 3 Aug 2022

[92] ICH. M4Q(R2) Common technical document on quality guideline (2021) Available from: https://database.ich.org/sites/default/files/ICH_M4Q-R2_ConceptPaper_Endorsed_2021_1115.pdf

[93] Robertson AS, Malone H, Bisordi F, Fitton H, Garner C, Holdsworth S, Honig P, Hukkelhoven M, Kowalski R, Milligan S, O’Dowd L, Roberts K, Rohrer M, Stewart J, Taisey M, Thakkar R, Van Baelen K, Wegner M (2020). Cloud-based data systems in drug regulation: an industry perspective. Nat Rev Drug Discov.

[94] Murali K, Kaur S, Prakash A, Medhi B (2019). Artificial intelligence in pharmacovigilance: practical utility. Indian J Pharmacol.

[95] Pfizer (2022) *AI in drug safety: building the elusive ‘Loch Ness Monster’ of reporting tools*. Available from: https://www.pfizer.com/news/articles/ai-drug-safety-building-elusive-%E2%80%98loch-ness-monster%E2%80%99-reporting-tools. Accessed 16

[96] Regulatory Affairs Professionals Society. RF Quarterly, Artificial intelligence (2022) December Available from: https://www.raps.org/news-and-articles/news-articles/2022/11/rf-quarterly-december-2022-artificial-intelligence. Accessed 14 Jan 2023

